# Diagnostic value of one-step nucleic acid amplification for sentinel lymph node metastasis in cytokeratin 19-positive tumors: evidence from bioinformatics and meta-analysis

**DOI:** 10.3389/fonc.2024.1370709

**Published:** 2024-04-08

**Authors:** Ke Li, Min Meng, Weiwei Zhang, Junyi Li, Yiting Wang, Changhui Zhou

**Affiliations:** ^1^ Department of Central Laboratory, Liaocheng People’s Hospital, Liaocheng, Shandong, China; ^2^ Department of Clinical Medicine, Shandong First Medical University, Jinan, Shandong, China

**Keywords:** OSNA, CK19, sentinel lymph nodes, metastasis, meta-analysis

## Abstract

**Background:**

The status of the sentinel lymph nodes (SLNs) was an important prognostic factor in varies cancers. A one-step nucleic acid amplification (OSNA) assay, a molecular-based whole-node analysis method based on CK19 mRNA copy number, was developed to diagnose lymph node metastases. We aimed to evaluate the value of OSNA for the diagnosis of sentinel lymph node metastasis in CK19 positive cancers. CK19 mRNA and protein expression for pan-caner analysis were obtained from TCGA and the Human protein atlas database.

**Methods:**

Two researchers independently searched the PubMed, Cochrane Library and Web of Science databases for qualified articles published before December 1, 2023. A meta-analysis was performed using MetaDisc and STATA. Risk bias and quality assessments of the included studies were evaluated, and a subgroup analysis was performed. Ten cancer types were found to be CK19 positively expressed and 7 of 10 had been reported to use OSNA for SLN detection.

**Results:**

After literature review, there were 61 articles included in the meta-analysis, which consisted of 7115 patients with 18007 sentinel lymph nodes. The pooled sensitivity and specificity of OSNA were 0.87 and 0.95 in overall patients. Moreover, we found the background CK19 expression in normal tissue affected the diagnostic accuracy of OSNA. In breast cancer, we performed subgroup analysis. OSNA exhibited to be a stable method across different population groups and various medical centers. In addition, when 250 copies/μl was chosen as the cutoff point of CK19 mRNA, there were a relatively higher sensitivity and AUC in detecting SLN micro-metastasis than 5000 copies/μl.

**Discussion:**

OSNA can predict the occurrence of SLN metastasis accurately in CK19 positive cancers, especially in breast cancer, colorectal cancer, lung cancer, gastric cancer and endometrial cancer. Our study warrants future studies investigating the clinical application of OSNA in pancreatic, ovarian and bladder cancers.

## 1 Introduction

Sentinel lymph nodes (SLNs) are the first regional LNs to which tumor cells metastasize through lymphatic vessels (1). The importance of tumor-associated lymphatic vessels and lymphangiogenesis in the formation of LNs metastasis has been emphasized and described during the last two decades (2). Lymphatic vessels from the tumor into LNs is thought to provide a route for metastatic cancer spread, which is prognostic of distant organ metastasis and poor survival (3). Once established in the SLNs, in most cases such as melanoma and breast cancer, tumor cells can migrate to non-SLNs in an orderly sequence. The analysis of SLNs consists of intraoperative evaluation and postoperative pathological examination (4). In terms of the postoperative evaluation, multistep formalin-fixed tissue sections stained by hematoxylin and eosin (H&E) with or without immunohistochemistry (IHC) are commonly used (5). In the intraoperative evaluation, frozen section (FS) and touch imprint cytology (TIC) are recommended (6). However, these methods all have some weaknesses, such as inaccuracy and their required time. Therefore, a novel and more efficient approach for intraoperative detection of SLN metastasis is urgently needed.

Cytokeratin 19 (CK19) belongs to a family of keratins, which are widely used as an epithelial marker in clinical practice and served as a useful research tool in diagnosis, management, and prognosis of the tumors (7). CK19 is reported to be overexpressed in a variety of epithelial malignancies because of its metastatic potential, such as gastrointestinal cancers, lung cancer and breast cancer (8). Recently, a one-step nucleic acid amplification (OSNA) assay, a molecular-based whole-node analysis method, was a rapid intraoperative molecular detection technique and developed to more sensitively diagnose lymph node metastasis via the quantitative measurement of target CK19 mRNA (9). The OSNA assay can quantify the total metastatic volume in a whole lymph node based on CK19 mRNA copy number (10). To date, numerous studies have focused on the diagnostic value of OSNA for detecting SLNs in different cancer types, but the results have been inconclusive. The anticipated drawbacks of OSNA method are false-negative results due to unstable CK19 expression.

In this study, we firstly explored the CK19 expression in different cancers and identified CK19 positive cancers. Next, we performed meta-analysis to investigate the diagnostic value of OSNA for detecting SLN metastasis in different cancers with CK19 positive expression. Our study provided evidence for patient selection when using OSNA in clinical practice.

## 2 Materials and methods

### 2.1 CK19 mRNA and protein expression data in pan-cancer

The expression data (mRNA-seq) were obtained from the Cancer Genome Atlas (TCGA) database (https://portal.gdc.cancer.gov/) was analyzed using TCGA-biolinks in R software (R×64 3.5.1). The raw data were integrated, CK19 expression in 31 cancer types including 9518 tumor samples and 5540 non-tumor samples was analyzed and compared with the normal tissue using the transcripts per million (TPM) values. Meanwhile, CK19 protein expression data in 20 cancer types containing 422 patients were obtained from the Human Protein Atlas database (http://www.proteinatlas.org/). The CK19 protein expression level was defined according to the results of IHC staining using anti-CK19 monoclonal antibody (Agilent Cat# M0888, RRID: AB_2234418).

### 2.2 Data sources and search strategy

Two investigators systematically and independently searched the PubMed, Cochrane Library and Web of Science databases for articles published before December 1, 2023. Quality studies were needed to provide information on the diagnostic accuracy of OSNA for SLN metastasis in patients with cancer. Subject terms used for the literature search included “(molecular intraoperative) AND cancer”, “(intraoperative molecular analysis) AND cancer”, “(intraoperative nucleic acid amplification) AND cancer”, “(intraoperative nucleic acid amplification) AND cancer”, “(intraoperative nucleic acid amplification) AND cancer”, “(intraoperative nucleic acid amplification) AND cancer”, “(one step nucleic acid amplification) AND cancer”, “(one-step nucleic acid amplification) AND cancer” and “(OSNA) AND cancer”. The range of the search was also extended to the reference lists of retrieved original and review articles. No further ethical approval is required since the program does not require the recruitment of patients and the collection of personal information.

### 2.3 Study selection

All articles were screened according to the inclusion and exclusion criteria by two independent reviewers. The inclusion criteria were as follows: (1) patients who were diagnosed with cancer; (2) the specimens collected were fresh SLNs; (3) the study’s purpose was to investigate the performance of the OSNA assay for detecting SLN metastasis in cancer patients; (4) the reference method for detecting SLN metastasis was postoperative pathology; (5) the study adopted identical machines and thresholds recommended by the OSNA manufacturer, Sysmex company; (6) the method of pathological examination was described in detail; (7) the study analysis was based on per node; and (8) extracted data were available for obtaining true-positive, false-positive, false negative, and true-negative values. The exclusion criteria were as follows: (1) non-English articles; (2) nonclinical research literature, including basic experiments, reviews, conference abstracts and letters to journal editors; and (3) intraoperative pathology, such as frozen section or touch imprint cytology (11).

### 2.4 Data extraction and synthesis

Two investigators independently evaluated the eligibility and quality of the studies. The data extracted were the first author, year of publication, country, type of study design, number of patients, number of lymph nodes, number of study centers, section interval, reference standard method, and type of samples. Diagnostic accuracy estimates included true positives (TPs), true negatives (TNs), false positives (FPs) and false negatives (FNs).

### 2.5 Assessment of diagnostic test accuracy

The diagnostic accuracy of OSNA for SLN metastasis was quantified by the area under the summary receiver operating characteristic (AUC), summary diagnostic odds ratio (DOR), summary sensitivity, specificity, positive likelihood ratio (PLR), negative likelihood ratio (NLR) and their 95% confidence interval (CI). A subgroup analysis was further performed to identify possible sources of heterogeneity.

### 2.6 Assessment of heterogeneity and publication bias

Heterogeneity was explored further by subgroup analyses. In addition, publication bias was assessed by the asymmetry of the Deek’s funnel plot (12). All statistical tests were two sided, and P < 0.05 was considered significant unless otherwise indicated. The above statistical analyses were completed with Stata 16.0.

## 3 Results

### 3.1 Selection of CK19 positively expressed cancers

**Figure 1:**
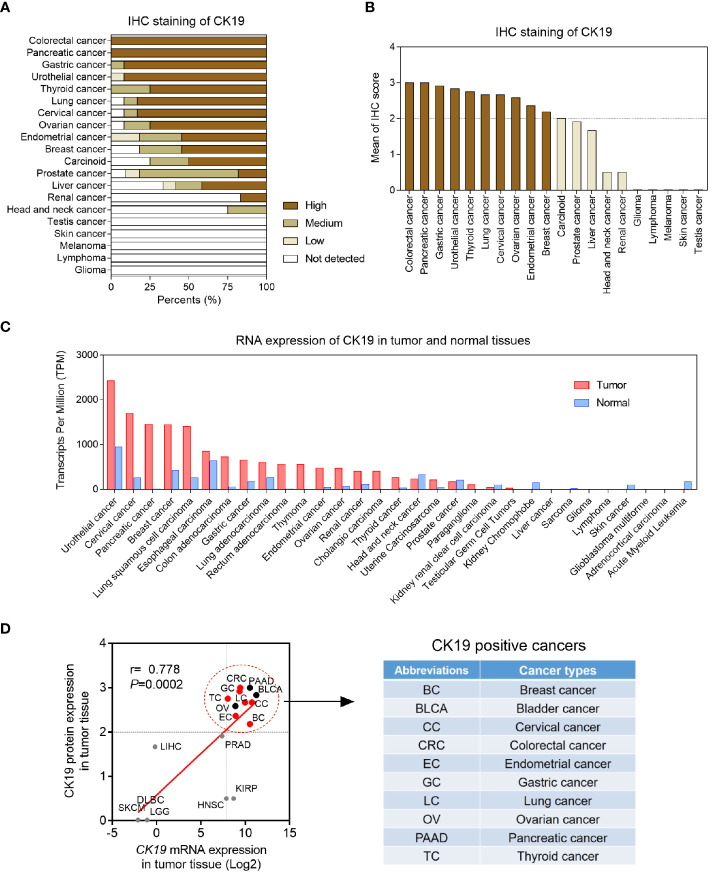
Pan-cancer CK19 protein and mRNA levels. **(A)** Pan-cancer CK19 protein levels based on IHC scores according to Human Protein Atlas (http://www.proteinatlas.org/). **(B)** Organized IHC scores of pan-cancer CK19 protein levels. High, medium, low and not detected protein levels were weighted as 3, 2, 1 and 0, respectively. Mean IHC score >2 was identified as high CK19 expressed tumors. **(C)** Pan-cancer and para-tumoral CK19 RNA levels according to TCGA (https://www.cancer.gov/). **(D)** Correlation CK19 mRNA expression and protein in different tumor tissues.

CK19 is well acknowledged as a biomarker of epithelial tumors. Tumor cells carrying high CK19 expression is found to be associated with high invasive phenotype. Therefore, we screened the CK19 protein expression in 20 cancer types (**Figure 1A**). The IHC staining results showed that the protein levels of CK19 were elevated in colorectal cancer, pancreatic cancer, gastric cancer, urothelial cancer, thyroid cancer, lung cancer, cervical cancer, ovarian cancer, endometrial cancer and breast cancer (mean IHC score >2) (**Figure 1B**). Considering the OSNA method is based on CK19 mRNA copy number, we next explored the mRNA expression of CK19 in 31 cancer types based on TCGA-sequencing data. The CK19 mRNA expression in tumor and normal tissues were presented in **Figure 1C**. After matching the mRNA expression and its corresponding protein expression in each cancer type, we found the cancer types with elevated CK19 IHC scores were always carrying high CK19 mRNA expression levels (r=0.778, P=0.0002). Finally, we identified ten cancer types, including breast cancer, bladder cancer, cervical cancer, colorectal cancer, endometrial cancer, gastric cancer, lung cancer, ovarian cancer, pancreatic cancer and thyroid cancer, as the CK19 positively expressed cancers (**Figure 1D**).

### 3.2 Characteristics of included studies for meta-analysis

**Figure 2:**
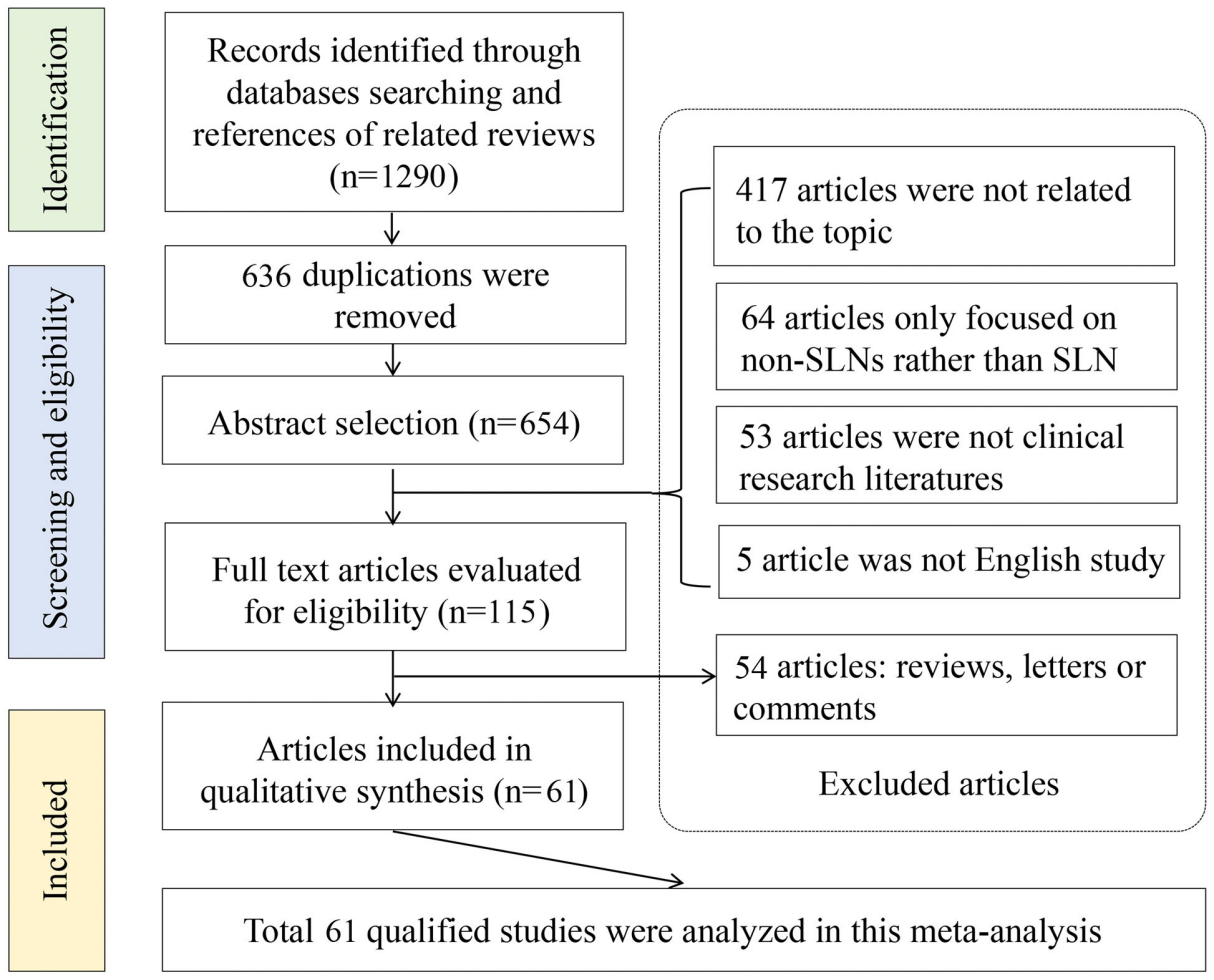
Flowchart of meta-analysis. A total of 1290 records were identified through databases, after screening, 636 articles were duplications. Six hundred and thirty-six articles were excluded, including 417 articles were not related to the topic, 64 articles only focused on the non-SLNs and 53 articles were not clinical studies. 54 articles did not have sufficient data for diagnostic testing. Finally, 61 articles were included in the meta-analysis.

Next, we investigated the diagnostic accuracies of OSNA in CK19 positive cancers using meta-analysis method. **Figure 2** is a flow chart that schematizes the exclusion of relevant articles for specific reasons. An initial search using predetermined key terms found 1290 potentially relevant articles, but 636 articles were duplications. Studies were also excluded because 417 articles were not related to the topic, 64 articles only focused on the non-SLNs and 53 articles were not clinical studies. 54 articles did not have sufficient data for diagnostic testing. In summary, 61 articles involving 7 cancer types were included in the meta-analysis (9, 10, 13–71). Four cancer types, including bladder cancer, head and neck squamous cell carcinoma, ovarian cancer and pancreatic cancer, were not included for meta-analysis, due to limited literature.

The basic characteristics of the 61 included studies are shown in **Table 1**. Our study consisted of 7115 patients with 18007 sentinel lymph nodes. Among them, 29 studies enrolled patients of breast cancer, 8 studies involving colorectal cancer, 7 studies involving thyroid cancer, 6 studies involving lung cancer, 4 studies involving gastric cancer, 4 studies involving endometrial cancer and 2 study enrolled patients of cervical cancer. The reference standards of all studies were assessed by postoperative pathology, but the detailed approaches were different, because 43 studies were taken with serial sections with HE staining and IHC and 15 studies undertook serial sections with HE staining only.

**Table 1:** Basic characteristics of all eligible studies in this meta-analysis.

Author(year)	Country	Cancer types	No. patients	No. SLNs	Reference method	TP	FP	FN	TN
Tsujimoto (2007) (9)	Japan	Breast cancer	49	81	HE and IHC	14	1	2	64
Visser (2008) (13)	Netherlands	Breast cancer	32	346	HE and IHC	61	15	3	267
Schem (2009) (14)	Germany	Breast cancer	93	343	HE and IHC	105	25	4	209
Tamaki (2009) (15)	Japan	Breast cancer	198	574	HE and IHC	89	25	11	449
Bernet (2011) (17)	Spain	Breast cancer	185	181	HE and IHC	42	1	0	138
Feldman (2011) (19)	America	Breast cancer	496	1044	HE and IHC	107	38	31	868
Khaddage (2011) (18)	France	Breast cancer	46	80	HE and IHC	15	1	2	62
Snook (2011) (20)	UK	Breast cancer	194	395	HE and IHC	66	10	6	313
Sun (2011) (21)	China	Breast cancer	90	189	HE and IHC	32	4	4	149
Goda (2012) (24)	Japan	Breast cancer	65	312	HE	53	10	8	241
Le Frère-Belda (2012) (26)	France	Breast cancer	234	503	HE and IHC	51	27	12	413
Wang (2012) (27)	China	Breast cancer	552	1188	HE and IHC	159	71	31	927
Buglioni (2013) (29)	Italy	Breast cancer	709	903	HE	174	28	14	687
Li (2013) (46)	China	Breast cancer	115	311	HE and IHC	30	9	6	266
Osako (2013) (31)	Japan	Breast cancer	80	307	HE	53	20	7	222
Sagara (2013) (32)	Japan	Breast cancer	53	61	HE and IHC	9	1	3	48
Wang (2014) (43)	Singapore	Breast cancer	NA	40	HE and IHC	19	0	1	20
Banerjee (2014) (33)	UK	Breast cancer	170	268	HE and IHC	39	10	2	217
Bettington (2014) (34)	Australia	Breast cancer	35	63	HE and IHC	9	3	1	52
Chaudhry (2014) (35)	UK	Breast cancer	54	166	HE and IHC	13	17	1	135
Jara-Lazaro (2014) (37)	Singapore	Breast cancer	54	98	HE and IHC	15	5	3	75
Pathmanathan (2014) (40)	Australia	Breast cancer	98	170	NA	25	5	3	137
Terada (2014) (41)	Japan	Breast cancer	89	111	HE	10	3	14	94
Hao (2014) (45)	China	Breast cancer	102	175	HE	39	13	9	113
Takamoto (2016) (51)	Japan	Breast cancer	88	300	HE and IHC	18	8	6	83
Shigematsu (2017) (56)	Japan	Breast cancer	499	1103	HE and IHC	104	26	30	943
Shimazu (2019) (63)	Japan	Breast cancer	63	150	HE	63	1	3	83
Inua (2021) (68)	UK	Breast cancer	691	684	HE	44	58	10	572
Pina (2022) (69)	France	Breast cancer	197	197	HE and IHC	30	44	10	113
Nagai (2015) (47)	Japan	Endometrial Cancer	35	137	HE	14	1	3	119
López-Ruiz (2016) (49)	Spain	Endometrial Cancer	34	94	HE and IHC	5	11	0	78
Fanfani (2018) (55)	Italy	Endometrial Cancer	40	110	HE and IHC	2	6	1	101
Kosťun (2019) (60)	Czech Republic	Endometrial Cancer	58	135	HE	10	18	1	106
Togami (2023) (66)	Japan	Cervical and endometrial cancer	133	437	HE and IHC	56	5	4	372
Okamoto (2013) (30)	Japan	Cervical cancer	32	130	HE and IHC	3	2	3	122
Bizzarri (2020) (64)	Italy	Cervical cancer	18	39	HE	6	2	1	9
González (2015) (44)	Spain	Thyroid cancer	5	50	HE and IHC	19	3	2	26
Kaczka (2014) (71)	Poland	Thyroid cancer	32	92	IHC	13	3	4	72
Kaczka (2015) (38)	Poland	Thyroid cancer	5	21	HE and IHC	9	2	0	10
del Carmen (2016) (48)	Spain	Thyroid cancer	37	284	HE and IHC	84	19	13	168
Kaczka (2017) (54)	Poland	Thyroid cancer	43	65	HE and IHC	20	5	3	37
Iglesias Felip (2019) (59)	Spain	Thyroid cancer	35	110	HE and IHC	25	17	0	68
Medas (2019) (61)	Italy	Thyroid cancer	13	26	HE and IHC	7	1	1	17
Yaguchi (2011) (22)	Japan	Gastric cancer	32	162	HE and IHC	40	4	5	113
Kumagai (2013) (39)	Japan	Gastric cancer	61	394	HE	45	14	9	326
Shimada (2020) (65)	Japan	Gastric cancer	43	439	HE	14	5	8	412
Gęca (2020) (70)	Poland	Gastric cancer	78	78	N/A	13	6	10	49
Croner (2010) (16)	Germany	Colorectal cancer	184	184	HE and IHC	37	5	3	139
Yamamoto (2011) (23)	Japan	Colorectal cancer	85	385	HE and IHC	79	7	4	295
Güller (2012) (28)	Switzerland	Colorectal cancer	22	313	HE	51	11	2	249
Vogelaar (2014) (42)	Netherlands	Colorectal cancer	NR	127	HE	23	20	5	79
Yamamoto (2016) (52)	Japan	Colorectal cancer	204	1925	HE and IHC	125	63	20	1717
Colling (2017) (53)	UK	Colorectal cancer	19	82	HE and IHC	13	2	1	66
Yeung (2017) (57)	UK	Colorectal cancer	16	78	HE and IHC	16	1	0	61
Esposito (2019) (58)	Italy	Colorectal cancer	17	34	HE and IHC	9	0	4	21
Inoue (2012) (25)	Japan	NSCLC	49	165	HE and IHC	19	1	1	144
Hayama (2014) (36)	Japan	Lung cancer	20	40	HE and IHC	4	3	0	33
Nakagawa (2016) (50)	Japan	NSCLC	111	410	HE and IHC	47	18	12	333
Escalante Pérez (2019) (62)	Spain	Lung cancer	160	705	HE and IHC	34	26	1	644
Namba (2022) (10)	USA	Lung cancer	105	214	HE	11	3	2	198
Miyoshi (2022) (67)	Japan	Lung cancer	58	199	HE and IHC	29	13	3	154

No., Number of; SLN, sentinel lymph nodes; HE, hematoxylin and eosin; IHC, immunohistochemistry; TP, true positive; TN, true negative; FP, false positive; FN, false negative; NA, not available.

### 3.3 Diagnostic accuracy of OSNA for SLNs in CK19 positive cancers

**Figure 3:**
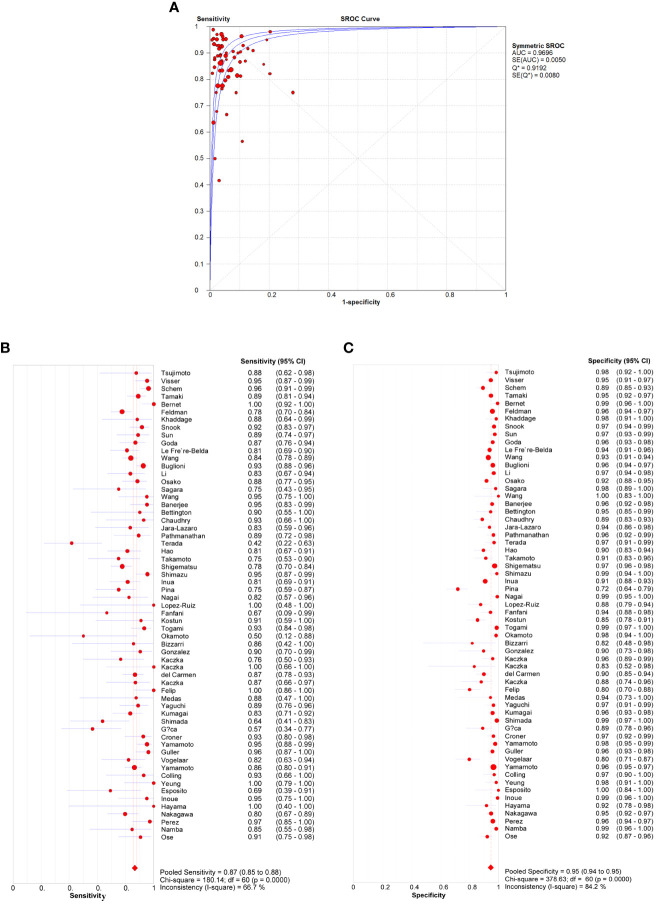
Diagnosis accuracy of OSNA through all studies. **(A)** ROC curve of OSNA by meta-analysis. The AUC was 0.9696. Sensitivity **(B)** and specificity **(C)** of each studies, pooled sensitivity and specificity were 0.87 and 0.95.

**Figure 4:**
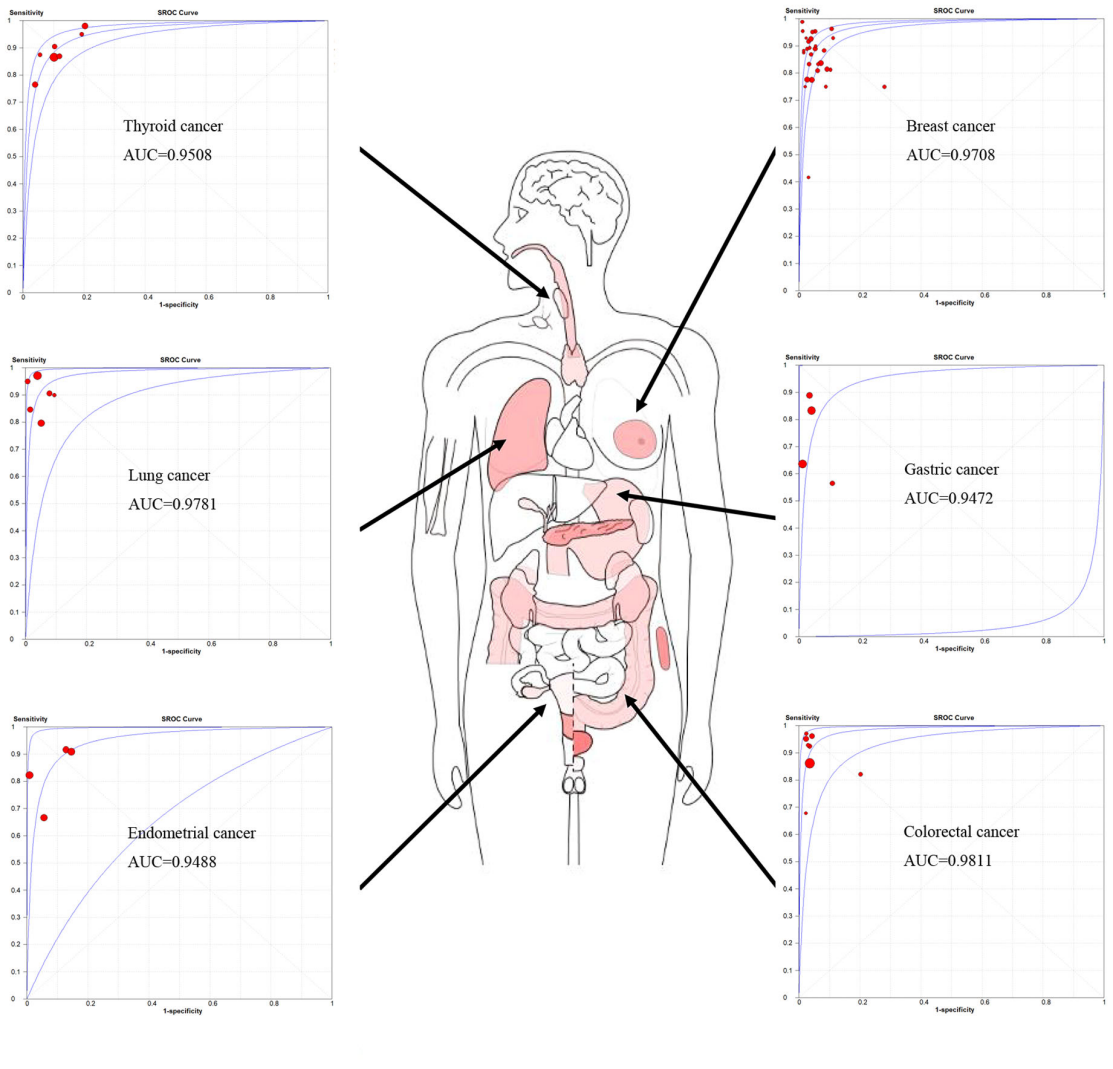
Landscape of OSNA diagnosis accuracy in different cancer types.

A total of 61 studies assessed the diagnostic accuracy of OSNA for SLNs. Overall, the pooled AUC of OSNA to diagnose SLN metastasis in CK19 positive cancer was 0.9696 (**Figure 3A**), with a sensitivity of 0.87 (95% CI from 0.85 to 0.88) and a specificity of 0.95 (95% CI from 0.94 to 0.95) (**Figures 3B, C**). Moreover, the diagnostic accuracy of OSNA for SLN metastasis was assessed across different cancer subtypes. The AUCs of thyroid cancer, breast cancer, lung cancer, gastric cancer, endometrial cancer and colorectal cancer were 0.9508, 0.9708, 0.9781, 0.9472, 0.9488 and 0.9811, respectively (**Figure 4**). In detail, the sensitivity for detecting SLN metastasis was highest in colorectal cancer (0.90, 95% CI=0.87-0.93) and lowest in cervical cancer (0.69, 95% CI=0.39-0.91). Meanwhile, the specificity was found to be highest in both cervical cancer (0.97, 95% CI=0.93-0.99) and gastric cancer (0.97, 95% CI=0.96-0.98). In contrast, the lowest specificity was shown in thyroid cancer (0.89, 95% CI=0.86-0.92) (**Table 2**).

**Table 2:** Diagnostic accuracies of OSNA in different cancer types.

Cancer types	Sensitivity	Specificity	PLR	NLR	DOR
Total	0.87 (0.86-0.88)	0.95 (0.95-0.96)	16.17 (13.46-19.42)	0.14 (0.12-0.18)	136.06 (104.84-176.58)
Thyroid cancer	0.89 (0.83-0.93)	0.89 (0.86-0.92)	7.35 (5.17-10.46)	0.15 (0.10-0.22)	65.45 (37.43-114.45)
Breast cancer	0.86 (0.85-0.88)	0.94 (0.94-0.95)	16.11 (12.21-21.25)	0.15 (0.11-0.19)	120.25 (80.95-178.63)
Lung cancer	0.88 (0.82-0.93)	0.96 (0.95-0.97)	20.09 (12.22-33.00)	0.12 (0.06-0.23)	218.44 (76.25-625.80)
Gastric cancer	0.78 (0.70-0.84)	0.97 (0.96-0.98)	19.19 (8.02-45.92)	0.26 (0.13-0.50)	78.86 (21.38-290.88)
Endometrial cancer	0.86 (0.71-0.95)	0.92 (0.89-0.94)	10.82 (4.48-26.10)	0.18 (0.09-0.38)	99.23 (29.06-339.56)
Cervical cancer	0.69 (0.39-0.91)	0.97 (0.93-0.99)	11.44 (1.67-78.62)	0.40 (0.16-0.99)	44.10 (8.48-229.30)
Colorectal cancer	0.90 (0.87-0.93)	0.96 (0.95-0.97)	21.51 (10.53-43.92)	0.11 (0.06-0.20)	234.03 (84.58-647.53)

OSNA, one-step nucleic acid amplification; PLR, positive likelihood ratio; NLR, negative likelihood ratio; DOR, diagnostic odds ratio.

### 3.4 Comparison of diagnostic accuracies and CK19 expression levels in seven cancer types

**Figure 5:**
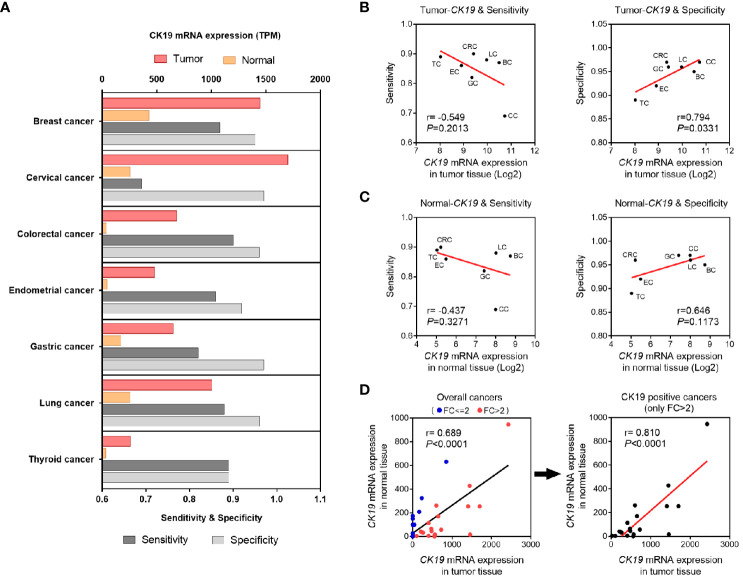
Association of CK19 expression and diagnosis accuracy of OSNA. **(A)** CK19 expression and diagnosis accuracy of OSNA in different cancer types. **(B, C)** OSNA diagnosis accuracy variants according to different CK19 expression levels in different types of tumor **(B)** or normal tissues **(C)**. **(D)** Correlation of CK19 mRNA expression in tumor tissues and normal tissues.

Considering the principle of OSNA method was based on CK19 mRNA copy number detection, we hypothesized that the diagnostic accuracy of OSNA might affected by CK19 mRNA expression in different cancer types. Therefore, we compared the sensitivity and specificity with CK19 mRNA expression, respectively (**Figure 5A**). Interestingly, we found that the sensitivity was negatively associated with tumor CK19 expression, whereas the specificity was positively associated with tumor CK19 expression (**Figure 5B**). Similarly, we observed a negative association between sensitivity and CK19 expression in normal tissue and a positive association between specificity and CK19 expression in normal tissue, indicating the background expression of CK19 might affect the diagnostic accuracy of OSNA (**Figure 5C**). Furthermore, we compared the background CK19 expression (normal tissue) and tumor CK19 expression. Our results confirmed that the background CK19 expression was elevated along with high expression of CK19 in tumor tissue, especially in CK19 positive cancers (**Figure 5D**).

### 3.5 Subgroup analysis of diagnostic accuracies of OSNA in breast cancer

Furthermore, we performed subgroup analysis in breast cancer, which included the largest number of studies (n=29). Our results showed a slightly difference of AUC among different subgroups, such as ethnic origins, patient number, LN number, center number, reference method and section interval (**Table 3**). Interestingly, we found the different OSNA cut-off values (250 or 5000 copies/μl) affected detecting micro-metastasis in breast cancer. In per-node micro-metastasis analysis, the sensitivity and specificity of 250 copies/μl were 0.69 and 0.95, respectively, whereas the sensitivity and specificity of 5000 copies/μl were 0.26 and 0.97, respectively. The diagnostic value of OSNA using 250 copies/μl to predict SLN micro metastasis (DOR, 44; AUC, 0.93) was substantially higher than using 5000 copies/μl (DOR, 16; AUC, 0.41). At the same time, there was no significantly difference between two cutoff values in diagnosing macro-metastasis. Thus, measurements of SLN micro-metastasis using 250 copies/μl may have a higher diagnostic accuracy.

**Table 3:** Subgroup analysis of diagnostic accuracies of OSNA in breast cancer based on a per-node analysis.

Subgroups	No.studies	No. Patients	No. Nodes	Sensitivity (95%CI)	Specificity (95%CI)	PLR (95%CI)	NLR (95%CI)	DOR (95%CI)	AUC (95%CI)
Ethnic origins									
Caucasian	9	1717	3185	0.93 (0.89-0.96)	0.95 (0.93-0.97)	19.8 (12.9-30.4)	0.07 (0.04-0.11)	290 (138-607)	0.98 (0.97-0.99)
Asian	15	2097	5000	0.84 (0.79-0.89)	0.96 (0.94-0.97)	19.0 (13.9-26.0)	0.16 (0.12-0.23)	115 (69-193)	0.97 (0.95-0.98)
Patient number									
≤100	15	989	2777	0.88 (0.82-0.93)	0.95 (0.93-0.97)	18.2 (13.1-25.3)	0.12 (0.08-0.19)	148 (85-255)	0.97 (0.96-0.98)
>100	11	3454	6645	0.88 (0.82-0.91)	0.96 (0.94-0.97)	20.6 (15.2-28.0)	0.13 (0.09-0.19)	158 (87-289)	0.98 (0.96-0.99)
LN number									
≤100	6	237	423	0.87 (0.79-0.93)	0.97 (0.93-0.98)	26.4 (13.2-52.9)	0.13 (0.08-0.23)	198 (77-507)	0.96 (0.93-0.97)
>100	21	4206	9039	0.88 (0.84-0.92)	0.95 (0.94-0.96)	18.5 (14.6-23.3)	0.12 (0.09-0.17)	151 (95-241)	0.97 (0.96-0.99)
Center number									
Single center	15	1746	3379	0.87 (0.81-0.91)	0.95 (0.94-0.97)	19.0 (14.3-25.2)	0.14 (0.09-0.21)	137 (81-232)	0.97 (0.96-0.98)
Multicenter	12	2697	6083	0.90 (0.84-0.94)	0.96 (0.94-0.97)	20.3 (13.8-29.7)	0.11 (0.07-0.17)	189 (94-377)	0.98 (0.96-0.99)
Reference method									
HE	4	416	730	0.88 (0.80-0.93)	0.95 (0.91-0.97)	16.2 (9.4-28.0)	0.13 (0.07-0.22)	129 (48-345)	0.97 (0.95-0.98)
HE+IHC	21	3964	8542	0.87 (0.82-0.91)	0.95 (0.94-0.96)	18.8 (15.0-23.7)	0.13 (0.10-0.18)	142 (94-215)	0.97 (0.96-0.98)
Interval									
≤2mm	19	2628	6002	0.88 (0.82-0.92)	0.96 (0.94-0.97)	19.7 (15.2-25.4)	0.12 (0.08-0.19)	158 (98-253)	0.98 (0.96-0.99)
Others	6	1623	6096	0.90 (0.84-0.93)	0.95 (0.93-0.97)	19.0 (12.9-27.9)	0.11 (0.07-0.17)	175 (81-380)	0.98 (0.96-0.99)
Micro-metastasis									
Cutoff =250	18	3813	7947	0.69 (0.64-0.74)	0.95 (0.95-0.96)	14.97 (7.28-30.77)	0.37 (0.28-0.49)	44 (26-76)	0.93 (0.90-0.95)
Cutoff =5000	4	276	1045	0.26 (0.13-0.42)	0.97 (0.96-0.98)	11.05 (3.09-39.52)	0.78 (0.66-0.93)	16 (4-66)	0.41 (0.35-0.46)
Macro-metastasis									
Cutoff =250	18	3813	7947	0.96 (0.95-0.97)	0.95 (0.95-0.96)	20.98 (10.28-42.82)	0.06 (0.04-0.08)	409 (225-743)	0.98 (0.98-0.99)
Cutoff =5000	4	276	1045	0.86 (0.79-0.92)	0.98 (0.96-1.00)	37.01(13.37-102.44)	0.14 (0.06-0.30)	326 (93-1145)	0.97 (0.97-0.98)

No., number of; 95%CI: 95% confidence interval; PLR, positive likelihood ratio; NLR, negative likelihood ratio; DOR, diagnostic odds ratio; AUC, the area under the summaryreceiver-operating characteristic curve; HE, hematoxylin and eosin staining; IHC, immunohistochemistry.

### 3.6 Heterogeneity and risk of publication bias

**Figure 6:**
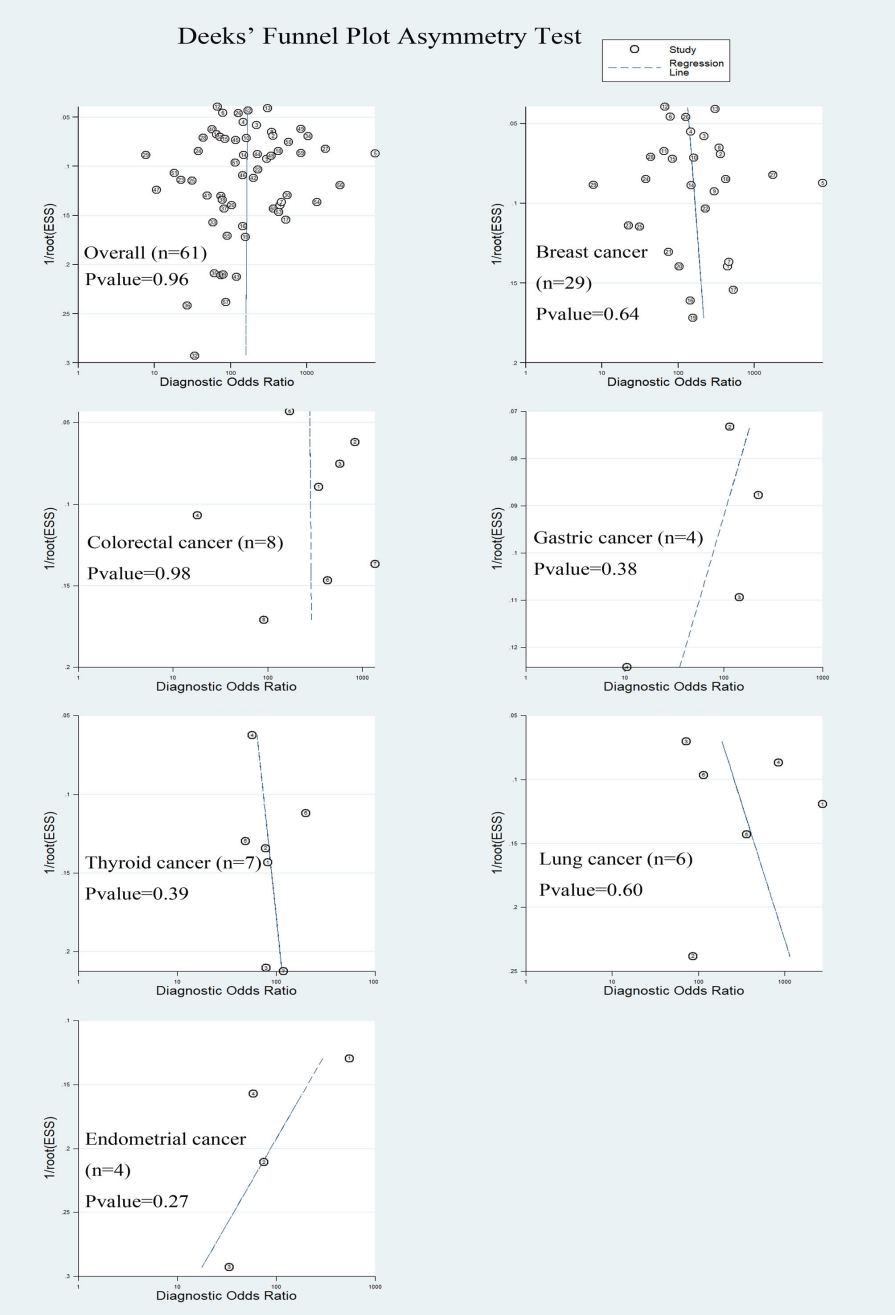
The Deeks’ funnel plot for assessing the publication bias.

The Deek’s funnel chart was used to analyze any potential publication bias. The results are shown in **Figure 6**. All funnel charts were symmetric, and P > 0.05, suggesting that no significant publication bias existed in this study.

## 4 Discussion

The formation of distant lymph node metastasis is the deadliest step of cancer progression and influence surgical decision making, which is an essential prognostic indicator in many different types of cancer (72). Therefore, LNM affects the prognosis and therapy of cancer patients in order to provide accurate assessment and effective treatment strategies (73). Classic intraoperative SLN detection methods, including FS and TIC, are limited due to a rather low sensitivity and no unified standardization. A meta-analysis based on intraoperative FS for SLNs suggested a pooled sensitivity of 0.73 (74). The poor sensitivity is related to the limited amount of tissue detected by FS and to the destructive, freezing, and compression artifacts of samples (75). To date, the diagnostic performance of OSNA for SLN metastases has been shown in a few studies. OSNA is shown to have a higher diagnostic performance than classic method. In addition, the turnaround time of the OSNA assay was less than 40 min for detecting one node, supporting OSNA to become a routine intraoperative SLN detection method. Nowadays, OSNA has been an established technique in breast cancer. In light of the high diagnostic performance in breast cancer, its accuracy for detecting SLN metastases in other CK19 positive cancers is not clear. To fill this void in knowledge, we attempted to conduct a meta-analysis to quantify the diagnostic accuracy of OSNA for the detection of SLN metastases in cancer patients carrying positive CK19 expression.

In this study, we firstly identified CK19 positive cancer by combining RNA sequencing and protein expression database. A total of 18 cancer types were shown to exhibit higher CK19 mRNA expression (fold-change >2), and CK19 IHC staining method confirmed 10 cancer types (mean IHC score >2). When performing literature review, we found OSNA method was reported to be used in seven cancer types, including breast cancer, cervical cancer, colorectal cancer, endometrial cancer, gastric cancer, lung cancer and thyroid cancer. However, three kinds of cancer, bladder cancer, ovarian cancer and pancreatic cancer, which also showed high CK19 expression and high capacities of LN metastasis, have no report of using OSNA in detecting LN metastasis. Therefore, our study suggested a pilot clinical study for evaluating the diagnostic accuracy of OSNA in above three cancers.

According to this meta-analysis, which included 61 related studies, we concluded that the pooled sensitivity, specificity and AUC of OSNA were 0.87, 0.95 and 0.97, respectively. By analyzing each subtype, we found the AUC of OSNA was higher than 0.95 in nearly all CK19 positive cancers. The high estimates suggested that OSNA appears to be a useful tool in assessing SLN metastasis for CK19 positive cancer patients. Next, we investigated that whether high CK19 expression would increase OSNA diagnostic accuracy. Interestingly, our data demonstrated that higher tumor CK19 mRNA expression increased specificity but decreased sensitivity of OSNA. We found that the CK19 expression in tumor tissues was strongly associated with its expression in normal tissues, indicating that a high background CK19 expression existed in CK19 positive cancers. Above findings might affect the diagnostic estimates of OSNA.

We selected breast cancer to perform subgroup analysis, mainly due to its largest study number. There is no significant difference between different subgroups, indicating that the OSNA was quite a stable method. We found that the cut-off values of OSNA affected its diagnostic accuracy to predict SLN micro-metastasis. Both sensitivity and AUC of OSNA using 5000 copies/μl were substantially lower than 250copies/μl. In clinical practice, the cut-off value of 250 copies/μl has been widely used for diagnosing micro-metastasis in breast cancer patients. However, we noticed that a few cancers, such as cervical cancer, had significantly higher CK19 expression than breast cancer. How to select appropriate cut-off values warrant further studies.

Given the potential benefits derived from the OSNA assay, OSNA is still proposed for use as a diagnostic tool in the intraoperative clinical setting. A reliable intraoperative SLN assessment is valuable for the optimal surgical treatment of breast cancer. The intraoperative analysis of the SLN is economically advantageous, and the implicit savings, resulting from the reduced number of hospitalizations and the avoidance of additional surgeries, compensates for the cost of the intraoperative OSNA assay (76). However, there are some limitations in our work. Firstly, we did not include studies with cancer patients receiving neoadjuvant chemotherapy and radiotherapy because of the insufficient data on this topic. Moreover, due to the lack of genotype information, we did not analyze the diagnostic values of OSNA in different cancer genotypes. Secondly, this meta-analysis mainly focused on the diagnostic value of OSNA, and its prognostic value could be evaluated in future studies (77).

In summary, this meta-analysis provides evidence that OSNA can predict the occurrence of SLN metastasis in patients with CK19 positive tumors. The diagnostic value of OSNA to predict SLN macro-metastasis was substantially higher than that of micro-metastasis. However, except for breast cancer, the number of studies involved in this analysis is limited, which may lead to insufficient evaluation. Therefore, these results need to be treated with caution, and should be further validated by more high-quality and multi-center clinical trials.

## Data availability statement

The original contributions presented in the study are included in the article/supplementary materials, further inquiries can be directed to the corresponding authors.

## Author contributions

KL: Writing – original draft, Writing – review & editing, Investigation. MM: Writing – original draft, Writing – review & editing, Conceptualization. WZ: Investigation, Resources, Writing – original draft. JL: Data curation, Project administration, Writing – original draft. YW: Conceptualization, Investigation, Supervision, Writing – review & editing. CZ: Conceptualization, Formal analysis, Investigation, Supervision, Writing – original draft, Writing – review & editing.
